# Recent Advances on the Multiplex Molecular Detection of Plant Viruses and Viroids

**DOI:** 10.3389/fmicb.2018.02087

**Published:** 2018-09-10

**Authors:** Vicente Pallás, Jesus A. Sánchez-Navarro, Delano James

**Affiliations:** ^1^Instituto de Biología Molecular y Celular de Plantas, IBMCP, Universitat Politècnica de València – Consejo Superior de Investigaciones Científicas, Valencia, Spain; ^2^Sidney Laboratory, Canadian Food Inspection Agency, Sidney, BC, Canada

**Keywords:** multiplex, polymerase chain reaction, molecular hybridization, microarrays, polyprobes, nextgeneration sequencing, plant viruses, viroids

## Abstract

Plant viruses are still one of the main contributors to economic losses in agriculture. It has been estimated that plant viruses can cause as much as 50 billion euros loss worldwide, per year. This situation may be worsened by recent climate change events and the associated changes in disease epidemiology. Reliable and early detection methods are still one of the main and most effective actions to develop control strategies for plant viral diseases. During the last years, considerable progress has been made to develop tools with high specificity and low detection limits for use in the detection of these plant pathogens. Time and cost reductions have been some of the main objectives pursued during the last few years as these increase their feasibility for routine use. Among other strategies, these objectives can be achieved by the simultaneous detection and (or) identification of several viruses in a single assay. Nucleic acid-based detection techniques are especially suitable for this purpose. Polyvalent detection has allowed the detection of multiple plant viruses at the genus level. Multiplexing RT polymerase chain reaction (PCR) has been optimized for the simultaneous detection of more than 10 plant viruses/viroids. In this short review, we provide an update on the progress made during the last decade on techniques such as multiplex PCR, polyvalent PCR, non-isotopic molecular hybridization techniques, real-time PCR, and array technologies to allow simultaneous detection of multiple plant viruses. Also, the potential and benefits of the powerful new technique of deep sequencing/next-generation sequencing are described.

## Introduction

Plant viruses and viroids are still a major concern in modern agriculture. They cause substantial economic losses in many important crops, especially those for which no virus-resistant varieties are available. Thus, early detection of these pathogens is still one of the main ways to control the development of the disease. Serology-based methods for virus detection have contributed significantly to evaluation of the sanitary status of these crops during the last 30 years and are still the methods of choice for a large number of laboratories involved in certification schemes (López et al., 2003; Boonham et al., 2014). The advent of nucleic acid-based technologies has allowed improving sensitivity to limits below the potential pathogenic thresholds solving some of the challenges posed by the need for specific and sensitive detection of plant viruses and viroids (Lopez et al., 2009). One of the most important challenges in plant virus/viroid diagnosis during the last decade has been the implementation of polyvalent and/or multiplex detection methods as these contribute to cost reductions, increased efficiency, and routine use (James et al., 2006). Different approaches, based on different biochemical principles, can be used to detect simultaneously multiple plant viruses or viroids including the following: (i) multiplex or polyvalent polymerase chain reaction (PCR), (ii) molecular hybridization including array techniques, and (iii) next-generation sequencing (NGS) technologies. The latter is revolutionizing the way plant virus diagnosticians are addressing the identification and characterization of new viruses and viroids and is having a profound impact on plant pathology in general (see Barba et al., 2014; Massart et al., 2014; Wu et al., 2015 for comprehensive reviews of NGS). However, PCR-based and molecular hybridization methods are still used frequently in most diagnostic laboratories due to years of validation, knowledge of their specificity and sensitivity, ease of implementation, and relatively low cost. In this short review, we update the progress made during the last 12 years on the multiplex or broad-spectrum detection of plant viruses and viroids.

## Molecular Hybridization

This technique is based on the complementarity of base pairs of nucleic acids that results in a stable hybrid formed by part (or the totality) of the nucleic acid sequence of the pathogen to be detected (target molecule), and the labeled complementary sequence (probe). Probes can be synthesized in the form of RNA (riboprobes) or DNA (DNA probes) molecules. Most plant viruses and all viroids have RNA as their genetic material. Since RNA–RNA hybrids are more stable than RNA–DNA or DNA–DNA hybrids and, consequently, more stringent conditions can be used with riboprobes detecting pathogenic RNAs, it is not surprising that riboprobes are the most frequent probes used in phytodiagnosis. In fact, molecular hybridization as a diagnostic tool in plant virology was first applied for the detection of viroids (Owens and Diener, 1981) for which no serological method could be used due to the lack of any protein component in their structural constituents. Subsequently, the technique was used for the detection of plant viruses (Garger et al., 1983; Maule et al., 1983). The stability of the resultant hybrid, and therefore the technical reliability, depend on both electrostatic and hydrophobic forces, which in turn depend on the reaction conditions such as temperature, salt concentration, and length of the probe, among other factors. Non-radioactive probes can detect RNA target molecules to the femtomole level when properly prepared and quantified. Detailed procedures for the synthesis of the labeled probe, sample preparation, hybridization, and detection have been described in previous reviews (e.g., Hull, 1993; Pallás et al., 1998, 2017; Mühlbach et al., 2003). Molecular hybridization has an intermediate sensitivity level between serological and PCR-based methods but maintains the user friendliness of the former and lacks the main disadvantages of the latter (higher possibility of false positives and contamination). It is not surprising that today more and more companies are increasingly offering molecular hybridization-based tests among their services to detect plant pathogens. As yet though there is no industry standard in terms of the format used. Although molecular hybridization is considered a very robust and reliable procedure, it is not exempt of some weaknesses. For instance, in some pathogen/host combinations, spurious hybridization signals can be observed due to host RNAs with sequence similarity with the plant virus/viroid to be detected (e.g., Cañizares et al., 1999). The potential false positives can be eliminated through incubation of the membranes with RNase at high ionic strength after hybridization. When molecular hybridization is applied in a tissue printing format, probes can bind to host proteins causing false positive signals as observed for the detection of peach latent mosaic viroid (PLMVd) in peach samples (WenXing et al., 2009). An extra step for removing proteins should be included to avoid inaccurate results (WenXing et al., 2009).

Remarkably, molecular hybridization can be easily adapted to simultaneously detect different viruses and/or viroids in a single hybridization assay (James et al., 2006). This can be addressed by mixing in the same probe solution different DNA or RNA probes or by synthesizing a unique probe that harbors in tandem the corresponding partial RNA (riboprobes) or DNA (DNA probes) complementary sequences to the plant viruses/viroids to be detected.

### Probe Mix

The first molecular hybridization approach used to detect several viruses at the same time involved the mixture of different probes, each specific for a different target, in the same assay. Probe mixtures have been successfully applied to phytosanitary certification of tomato in Italy (Saldarelli et al., 1996), to the simultaneous detection of five viruses affecting carnation (Sánchez-Navarro et al., 1999), three ilarviruses affecting stone fruit trees (Saade et al., 2000), two viruses affecting geranium plants (Ivars et al., 2004), and of 10 artichoke viruses (Minutillo et al., 2012) (**Table 1**). In all these cases, probes (DNA or RNA) were mixed in the hybridization solution and reliably detected all target viruses, with high specificity and identical sensitivity to that obtained using individual probes. The main disadvantage of this approach is that mixtures of many riboprobes can result in undesirable background that can make the results indecipherable. Treating the membranes with RNase A after the washing steps can overcome, at least in part, this drawback (Sánchez-Navarro et al., 1999). In addition, this disadvantage can be totally overcome by the use of a unique probe, polyprobe, containing in tandem several partial sequences of different viruses and/or viroids (see next section). A mixture of two DNA probes has been recently used that allowed the simultaneous visualization of two Citrus tristeza virus genotypes in vascular and non-vascular tissues of citrus trees (Bergua et al., 2016). This approach can be of great interest to study the basis for the interactions between different components of virus populations and for getting a deeper knowledge of the superinfection exclusion phenomenon at the cellular level.

**Table 1:** Polyvalent molecular hybridization assays for the detection of plant viruses and viroids.

Probe type	Crop applied or virus type	No. of targets	Nature of targets	Reference
Mixed riboprobes	Tomato	6	Viruses	Saldarelli et al., 1996
Mixed riboprobes	Carnation	5	Viruses	Sánchez-Navarro et al., 1999
Mixed riboprobes	Stone fruits	3	Viruses	Saade et al., 2000
Mixed riboprobes	Geranium	2	Viruses	Ivars et al., 2004
Mixed DNA probes	Artichoke	10	Viruses	Minutillo et al., 2012
Polyprobe	Stone fruits	6	Viruses	Herranz et al., 2005
Polyprobe	Citrus	3	Viroids	Cohen et al., 2006
Polyprobe	Tomato	6	Viruses	Aparicio et al., 2009
Polyprobe	Pome and stone fruits	6	Viroids	Lin et al., 2011
Polyprobe	Stone fruits	10	8 Viruses + 2 viroids	Peiró et al., 2012
Polyprobe	Grapevine	4	Viroids	Zhang et al., 2012
Polyprobe	Ornamentals and vegetables	8	Viroids	Torchetti et al., 2012
Polyprobe	Tomato	3	1 virus + 1 viroid + 1 bacteria	Zamora-Macorra et al., 2015
Polyprobe	Coleus	8	Viroids	Jiang et al., 2013
Polyprobe	Apple and pear	4	Viruses	Fajardo and Nickel, 2014
Polyprobe	Grapevine	18	13 Viruses + 5 viroids	Sánchez-Navarro et al., 2018b
Polyprobe	*Potyvirus* genus	32	Viruses	Sánchez-Navarro et al., 2018a
Arrays	Potato	6	Viruses	Boonham et al., 2003; Bystricka et al., 2003
Arrays	Cucurbits	4	Viruses	Lee et al., 2003
Arrays	Fruit trees	7	Viruses	Lenz et al., 2008
Arrays	Potyviruses	4	Viruses	Wei et al., 2009
Arrays	Tomato	10	Viruses	Tiberini et al., 2010
Arrays	Tomato	16	Viruses + viroids	Tiberini and Barba, 2012
Arrays	Artichoke	14	Viruses	Tiberini and Barba, 2013
Arrays	Grapevine	44	Viruses	Engel et al., 2010
Arrays	Grapevine	15	Viruses	Abdullahi et al., 2011
Arrays	General	52	Viruses	Nicolaisen, 2011
Arrays	General	37	Viroids	Zhang et al., 2013


### Polyprobes

This approach, developed in 2005 by Herranz et al. (2005) uses a unique polyprobe for the polyvalent detection of different pathogens in a single assay. A polyprobe results from the cloning, in tandem, of complementary partial sequences, usually between 200 and 400 nucleotide residues, of different viruses/viroids after the promoter region of a RNA polymerase. After digestion with the appropriate restriction enzyme, a long transcript is synthesized in the presence of a non-radioactive precursor. Hybridization and immunological detection steps have been previously explained in detail (e.g., Pallás et al., 1998; Mühlbach et al., 2003) and will not be described here.

Polyprobes have been applied successfully for the detection of an array of combinations of plant viruses and/or viroids affecting different crops (**Table 1**). Polyprobes have been developed and used to detect four viroids affecting citrus trees (Cohen et al., 2006), the six main viruses infecting tomato (Aparicio et al., 2009), six viruses affecting pome and stone fruits (Lin et al., 2011), four viroids affecting grapevine (Zhang et al., 2012), eight viroids affecting ornamentals and vegetables (Torchetti et al., 2012), eight viroids infecting coleus plants (Jiang et al., 2013), and four viruses of apple and pear trees (Fajardo and Nickel, 2014). Remarkably, this technology has great potential for simultaneous multipathogen detection. Using polyprobes, Peiró et al. (2012) were able to detect eight viruses and two viroids infecting stone fruit trees. In addition, three pathogens with very different life cycle styles (bacteria, virus, and viroid) were simultaneously detected in a single assay in tomato plants (Zamora-Macorra et al., 2015). In general, the polyprobes permit the detection of several pathogens with comparable detection limit to the individual probes, although with long polyprobes (e.g., 10 probes in tandem or more) a reduction of the hybridization temperature is required (e.g., Peiró et al., 2012). In our hands, up to 18-mer polyprobes (detecting 13 viruses plus 5 viroids of grapevine; Sánchez-Navarro et al., 2018b) gave clear and reliable results without loss of sensitivity. Recently, a unique polyprobe with the capacity to detect all members of the *Potyvirus* genus was developed (Sánchez-Navarro et al., 2018a). The authors observed that sequences of the different potyvirus species showing a percentage similarity of 68% or higher, could be detected with the same probe by hybridizing at 50–55°C, with a detection limit of picograms of viral RNA comparable to the specific individual probes. The developed polyprobe contains seven different 500-nt fragments of a conserved region of the NIb gene and was able to detect all 32 potyviruses assayed with no signal in the healthy tissue. This assay is being considered as a genus probe for general potyvirus detection.

### Arrays

Another broad-spectrum approach for parallel detection of multiple plant viruses relies on the DNA microarray technology. The technical details as well as the historical concept and development of DNA microarrays have been described exhaustively in previous reviews (Hadidi et al., 2004; Boonham et al., 2007; Zhu et al., 2017). Due to the high-throughput nature of the array technology, this approach was expected to have, in principle, a great potential for broad-spectrum diagnostics of plant viruses and viroids. However, the application of this technology has been limited so far. This is mainly due to the relative complexity of the different steps required to accomplish implementation of the test. This approach was first applied for the detection and identification of six potato viruses (Boonham et al., 2003; Bystricka et al., 2003, 2005) and four species of selected cucurbit-infecting tobamoviruses (Lee et al., 2003). An oligonucleotide microarray for the detection of some fruit tree viruses was designed and its theoretical detection limit was assessed (Lenz et al., 2008). The authors concluded that the sensitivity of detection is, among others, influenced by the proximity of the probe hybridization site to the unlabeled end of the targets. Wei et al. (2009) developed a 25-mer oligonucleotide microarray targeting four distinct potyviruses that included 85 probes designed from conserved and variable sequence regions of the nuclear inclusion b (NIb) gene, RNA-dependent RNA polymerase (RdRp) gene, coat protein (CP) gene, and the 3′ untranslated region (UTR), specific to the four targeted potyviruses at both species and strain levels. Using “Combimatrix” platform 40-mer oligonucleotide probes, Tiberini et al. (2010) designed a DNA microarray chip for screening 10 major economically important tomato viruses and later on this platform was optimized to include six pospiviroid species (Tiberini and Barba, 2012). This same platform was used to develop an oligonucleotide-based microarray for detection of multiple artichoke viruses (Tiberini and Barba, 2013). This diagnostic array demonstrated its applicability for routine diagnostic use in artichoke germplasm as it detected simultaneously 14 viruses in one single hybridization event. Two oligonucleotide microarrays have been developed for detecting grapevine viruses. Engel et al. (2010) used a 70-mer microarray containing 570 unique probes designed against highly conserved and species-specific regions of 44 grapevine viral genomes, whereas Abdullahi et al. (2011) used a range between 27 and 75 nucleotides in length for oligonucleotides and detected eight nepoviruses, two vitiviruses, and one each of closterovirus, foveavirus, ampelovirus, maculavirus, and sadwavirus. Nicolaisen (2011) developed a microarray with 150 probes potentially capable of detecting 52 viruses from a broad range of genera. Forty nine of the 52 species tested were identified correctly to species level. Finally, Zhang et al. (2013) designed a microarray with a minimal number of probes that can detect a wide spectrum of all 8 reported viroid genera including 37 known plant viroid species.

Microarrays can be used not only for diagnostics but for phylogenetic or taxonomic purposes. An oligonucleotide-based microarray was developed to detect and differentiate cucumber mosaic virus (CMV) serogroups and subgroups (Deyong et al., 2005). A long 70-mer oligonucleotide DNA microarray was developed that was capable of simultaneously detecting and genotyping plum pox virus strains (Pasquini et al., 2008).

## Multiplex PCR

**FIGURE 1:**
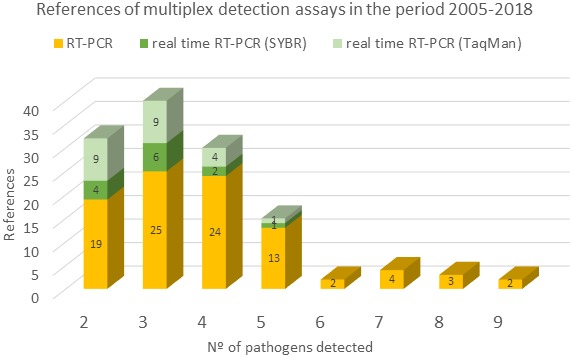
References of multiplex detection assays in the period 2005–2018. Number of references that use RT-PCR (yellow), real-time RT-PCR SYBR (green), and real-time RT-PCR TaqMan (light green) are represented against the number of pathogens detected by these techniques. Source used was the Web of Science (all database) and the parameters used for the searching were: (multiplex or simultaneous or polyvalent) and detection and (plant virus or viroid).

Multiplex PCR (DNA targets) and multiplex RT-PCR (mRT-PCR) (RNA targets) are quick, reliable, and cost-effective methods that have been used successfully for detecting a variety of pathogens simultaneously in a single assay. Whereas uniplex RT-PCR is potentially expensive and resource intensive (requiring time and resources to test for each virus or viroid separately), mRT-PCR incorporates different sets of specific primers for two to more targets in one reaction tube and enables simultaneous amplification of different target nucleic acids in a single test. This reduces material costs, labor, and time. It is indeed possible also to amplify simultaneously several regions of a target virus thereby improving the reliability of detection. The main approach for this purpose is the mRT-PCR which uses specific or degenerative primers that amplify and allow identification of the size and species-specific amplicons by agarose gel analysis. This approach represents 71.8% of all multiplex detection reports since 2005 (**Figure 1**), allowing the simultaneous detection of as many as six (Park et al., 2005; Cating et al., 2015), seven (Roy et al., 2005; Tao et al., 2012; Kwon et al., 2014; Zhao et al., 2015), eight (Sánchez-Navarro et al., 2005; Deb and Anderson, 2008; Kwak et al., 2014), and up to nine (Gambino and Gribaudo, 2006; Gambino, 2015) pathogens. However, the sensitivity of this technique is influenced by the number of targets to be detected (Sánchez-Navarro et al., 2005; Tao et al., 2012; Kwon et al., 2014; Nam et al., 2015), mainly due to the number of different primer pairs instead of the total amount of primer present in the cocktail. Sánchez-Navarro et al. (2005) showed that the use of a cocktail of five primer pairs do not affect the detection limit of mRT-PCR, while seven pairs does affect the detection limit. In agreement with this observation, 91.4% of the multiplex reactions reported since 2005 describe the detection of two to five pathogens (**Figure 1**). Some of these limitations have been overcome by improving the quality of the nucleic acid extraction procedures, the products obtained and/or different modalities of the PCR technology. Magnetic nanobeads (Deng et al., 2014), dual priming oligonucleotide (DPO) primers (Kwon et al., 2014), or a nested reaction (Foissac et al., 2005; Maliogka et al., 2007; Papayiannis et al., 2011) have all contributed to increased levels of specificity and sensitivity.

Other limitations of the mRT-PCR reaction are the use of the agarose gel-based detection for discriminating the size-specific amplicons with its putative optical error and the confirmation that the amplified DNA fragments correspond to the target sequences. Some alternative approaches have eliminated the use of agarose gels such as the use of species-specific biotinylated probes in streptavidin-coated microtiter wells (Charoenvilaisiri et al., 2014) or, more recently, the use of a platform for multiplexed nucleic acid detection such as the microsphere-based flow cytometric system developed by Luminex (Austin, TX, United States). The Luminex xMAP system incorporates 5.6 μm polystyrene microspheres that are internally dyed with two spectrally distinct fluorochromes. Using precise amounts of each of these fluorochromes, an array is created consisting of 100 different microsphere sets with specific spectral addresses. Each microsphere set can possess a different reactant on its surface (e.g., a unique anti-MagPlex-TAG oligonucleotide sequence). Because microsphere sets can be distinguished by their spectral addresses, they can be combined, allowing up to 100 different targets to be measured simultaneously in a single reaction vessel. A third fluorochrome coupled to a reporter molecule quantifies the biomolecular interaction that has occurred at the microsphere surface. Microspheres are detected individually in a rapidly flowing fluid stream as they pass by two separate lasers in the Luminex^®^ 100^TM^ analyzer. Thousands of microspheres per second could be detected, resulting in an analysis system capable of analyzing and reporting up to 100 different reactions in a single reaction vessel in just a few seconds per sample. The LUMINEX bead-based array has been applied for the multiple detection of begomovirus (van Brunschot et al., 2014a), pospiviroids (van Brunschot et al., 2014b), and lily viruses (Lim et al., 2016). This luminex approach implies the amplification of the target sequences by mRT-PCR reaction, which could be a bottleneck if many primers should be used, as commented above. Although this technology has been successfully used to detect related pathogens by using universal primers in the RT-PCR reaction (e.g., begomovirus, pospiviroids), it now remains to know if this technology could be adjusted to detect many unrelated pathogens.

Another alternative to the analysis of the amplicons by agarose gel-based detection, is the multiplex real-time PCR in which, the amplified fragments are detected directly during the reaction by using non-probe based fluorescent dyes such as SYBRGreen or EvaGreen (Zipper et al., 2004; Mao et al., 2007) or specific fluorescent probes such as TaqMan^®^ probes (Livak et al., 1995), molecular beacons (Tyagi and Kramer, 1996), or the minor groove binding (MGB) probes (de Kok et al., 2002). Real-time PCR allows quantification of the pathogen and reduced significantly the detection limit to as little as a few molecules (Torres et al., 2005; Mortimer-Jones et al., 2009; Tuo et al., 2014; Huo et al., 2015). However, despite the clear advantages of the real-time procedure, the references of multiplex detection assays based on this technique, either using non-probe fluorescent days or fluorescent probes, represent only 28.1% of such test since 2005 (**Figure 1**). The tests have been adapted to allow the simultaneous detection of two to five pathogens. The problems derived from the necessity of discriminating between different labeled amplicons by melting curve analysis or the availability of fluorescent dyes with overlapping excitation/emission spectra, have hampered the number of pathogens detected by multiplex real-time PCR. The few multiplex real-time PCR assays that use melting curve analysis based on the SYBR Green I, detect three targets at most, due to the shortcomings of the dye (Guion et al., 2008). This problem has been overcome by using the EvaGreen or SYTO dyes (Eischeid, 2011), which are less inhibitory towards PCR and provide better peak resolution, allowing the detection of up to five viruses (Bester et al., 2012; Cheng et al., 2013; Aloisio et al., 2018).

Multiplex detection of three or more targets by fluorescent probes is influenced by the availability of dyes with compatible excitation/emission spectra. Simultaneous detection of four pathogens has been reported for four retroviruses (Vet et al., 1999) or four potato (Agindotan et al., 2007; Mortimer-Jones et al., 2009) or cassava (Otti et al., 2016) viruses. In addition, this technology was adapted for the detection of five grapevine viruses, although it was necessary to perform a compensation color assay, which aimed to minimize the emission interference among the five fluorescent dyes (Lopez-Fabuel et al., 2013).

Multiplex RT-PCR tests have also been developed to detect all members of a specific genus by designing degenerative or specific primers that target conserved regions (**Table 2**). Obviously, a previous step for using this approach is the identification of conserved genomic regions that can be targeted by the amplification primers. Thus, this procedure has allowed the detection of virus species in the genera *Allexvirus* (Kumar et al., 2010; Majumder and Baranwal, 2014), *Ilarvirus* (Maliogka et al., 2007), *Bromovirus* and *Cucumovirus* (Seo et al., 2014), *Begomovirus* (van Brunschot et al., 2014a), *Potyvirus* (Zheng et al., 2010), and members of the viroid genus *Pospiviroids* (Botermans et al., 2013; Luigi et al., 2014; Olivier et al., 2014; van Brunschot et al., 2014b) or *Trichovirus*, *Capillovirus* and *Foveaviru*s (Foissac et al., 2005), or viroids and phytoplasmas (Malandraki et al., 2015). In the majority of cases, the species-specific identification was performed by restriction fragment length polymorphism (RFLP) analysis or by sequencing the corresponding amplicons. However, the introduction of the Luminex technology opens an interesting tool to allow the direct identification of the pathogen without any further analysis (van Brunschot et al., 2014a,b).

**Table 2:** Polyvalent PCR assays for the detection of plant viruses and/or viroids at the genus level.

Genus	Primers	Region	Amplicon (bp)	Species identification	Reference
Ilarvirus	Degenerated	RNA2/RdRp gene	381	Amplicon sequencing/RFLP	Maliogka et al., 2007
Pospiviroid	Degenerated		270	Luminex	van Brunschot et al., 2014b
Pospiviroid	Specific/several primers	Terminal conserved region (TCR) and terminal right domain (TR)	170–180	TaqMan probe only for genus	Botermans et al., 2013
Bromovirus/cucumovirus	Degenerated with adaptor sequences	RNA1	337	Amplicon sequencing	Seo et al., 2014
Pospiviroid	Degenerated		200	Amplicon sequencing	Olivier et al., 2014
Pospiviroid	Degenerated	The terminal left and the pathogenesis domains	300	Amplicon sequencing/RFLP	Luigi et al., 2014
Begomovirus	Degenerated	C3 ORF	290	Luminex	van Brunschot et al., 2014a
Potyvirus	Degenerated	NIb	350	Amplicon sequencing	Zheng et al., 2010
Trichoviruses, capilloviruses, foveaviruses	Degenerated	RdRp	362	Amplicon sequencing	Foissac et al., 2005
Phytoplasmas	Specific	16S rDNA	200	TaqMan only for phytoplasmas	Christensen et al., 2013; Malandraki et al., 2015
Allexivirus	Degenerated	3′-end of ORF6	183–192	Amplicon sequencing	Kumar et al., 2010; Majumder and Baranwal, 2014

## Multiplexing Potential of Next-Generation Sequencing

Next-generation sequencing, known also as massively parallel sequencing or deep sequencing, is a powerful technology that allows the generation of massive amounts of sequence data. There are various approaches or NGS platforms, each with different characteristics and with the potential in some cases to generate as many as three billion reads per run with read lengths that vary from approximately 35 to 800 nucleotides depending on the platform (Barzon et al., 2011; Buermans and den Dunnen, 2014; Wu et al., 2015; Jones et al., 2017; Rott et al., 2017). The technology has been proposed as a valuable tool for diagnostic virology (Adams et al., 2009; Barzon et al., 2011). NGS is highly sensitive and has the potential to detect the full spectrum of viruses infecting a given host, including known and even unknown viruses (Barzon et al., 2011; Villamor et al., 2016; Rott et al., 2017; Jo et al., 2018). The broad-spectrum and unbiased nature of the technology makes it a valuable tool for plant-based metagenomics, allowing the simultaneous screening and detection of populations of graft transmissible agents that include viruses (RNA and DNA), viroids, and phytoplasma in a sample (Adams et al., 2009; Kreuze et al., 2009). Jones et al. (2017) indicated that the multiplexing potential of NGS (RNA-seq) might allow a researcher to answer the question of how many different viruses are present in a crop plant. NGS even has the power to detect plant viruses that were not detected using current and standard tools that were based on biological, serological, and molecular tests (Villamor et al., 2016). See Jones et al. (2017) for an excellent review describing the use of RNA-seq for the detection of multiple viruses in each of various host plants.

Kreuze et al. (2009) used NGS to simultaneously detect/identify the viruses sweet potato feathery mottle virus (SPFMV, family *Potyviridae*), and sweet potato chlorotic stunt virus (family *Closteroviridae*). They targeted small RNAs (sRNA, 20–24 nt) in total RNA extracts from co-infected plants. Using this approach, they were able to assemble the complete genome of SPFMV and detected unexpectedly also two new and distinct badnaviruses (family *Caulimoviridae*) and a new mastrevirus (family *Geminiviridae*). This approach allowed the simultaneous detection of viruses with RNA and DNA genomes and viruses from distinctly different families. Verdin et al. (2017) used also a similar strategy targeting small RNAs from a range of ornamental plants, with pooling of samples, to detect (+) and (–) ssRNA viruses, dsRNA viruses, dsDNA viruses, an ssDNA virus, and a viroid. NGS allows simultaneous detection and identification of viruses belonging to different families and genera, but also multiple isolates or variants of the same virus co-infecting a single host. James et al. (2017) detected, by NGS analysis of total RNA extracted from a single apple plant, an infection complex that included apple chlorotic leaf spot virus (ACLSV, genus *Trichovirus*) and apple stem pitting virus (ASPV, genus *Foveavirus*). Five isolates/variants of ACLSV as well as 14 definite (but perhaps as many as 29) isolates/variants of ASPV were identified, all in a single sample. Rott et al. (2017) described the detection of 12 distinct genotypes of ASPV in sample #103 of their analyses. This shows the incredible ability of NGS to allow simultaneous detection and differentiation even of similar and closely related genomic sequences, which is not easily achieved with other diagnostic tools. Multiplex detection that allows accurate identification of variants or isolates of a single virus present in a sample may be desirable in some circumstances as this may have biological significance, influencing disease symptoms (James et al., 2017).

The nucleic acid template used for NGS analysis influences the reliability of simultaneous and broad-spectrum detection of plant viruses. Total RNA and small RNAs are effective targets for broad spectrum and even simultaneous detection of DNA viruses, RNA viruses and viroids (Kreuze et al., 2009; Wylie et al., 2012; Massart et al., 2014; Zhang et al., 2014; Wu et al., 2015; Verdin et al., 2017). Wu et al. (2015) suggest that only NGS analysis of total small RNAs is suitable for simultaneous detection of DNA viruses, RNA viruses, and viroids. For broad-spectrum virus detection Wylie et al. (2012) used a strategy of pooling total RNA from several plants, targeting polyadenylated RNA by using oligo-d(T) primers and was able to simultaneously detect by NGS 16 virus species belonging to various genera. Double-stranded (ds) RNA, the replicative form of RNA viruses (Dodds et al., 1984), as a template for NGS may be limited in that it may allow only reliable detection of RNA viruses and viroids (Massart et al., 2014). However, in comparing total nucleic acid extracts (TNA) to dsRNA for the analysis of infected grapevine, Al Rwahnih et al. (2009) obtained 54,605 viral hits from the dsRNA template versus 1,275 viral hits for the TNA template. If the targets for detection are known RNA viruses, perhaps the use of dsRNA for analysis will improve sensitivity. Jo et al. (2018) indicated that mRNA targets enriched by oligo dT were suitable for the detection and identification of different types of viral genomes including DNA viruses, ds RNA viruses, and viroids. The authors suggested that (U)-rich regions in viruses without a poly-A tail can be amplified by oligo dT.

As with any technology, there are concerns and/or limitations associated with the use of NGS as a diagnostic tool for plant viruses. There is a need for suitable bioinformatics tools and expertise to extract the required information from the enormous amounts of data generated (Wu et al., 2015; Visser et al., 2016; Roossinck, 2017). Small or low-resourced research groups may not always have access to bioinformaticians or access to a bioinformatics facility and this may influence their ability to utilize effectively the technology (Jones et al., 2017). The high sensitivity of NGS makes it susceptible to cross contamination including contamination with samples containing mycoviruses and insect viruses (Rott et al., 2017). To minimize the occurrence of false positives by NGS analysis, it is proposed that at least two different approaches be used for virus detection and identification (Rott et al., 2017; Jo et al., 2018). On the other end of the sensitivity spectrum, Jo et al. (2018) reported that NGS did not detect viruses or viroids in low titer that could be detected by RT-PCR. There is the possibility that virus sequences detected by NGS may be the remnants of sequences incorporated into host genomes (Martin et al., 2016); also, the biological significance of novel viruses or in some cases partial sequences detected need to be determined. There is a need for validation data, for more information on the sensitivity of NGS-based detection, compared to established diagnostic techniques such as real-time RT-PCR, and the definition of thresholds for a positive detection are needed (Massart et al., 2014).

In their review of various publications describing NGS analysis for plant virus detection, Jones et al. (2017) identified a number of issues, at least two of which have special significance for reliable diagnosis. These include the detection of different viruses or levels of viruses associated with different parts of the plants and the fact that different analytical tools can give different results for viruses being detected. Consistent and appropriate sampling and the choice of appropriate analytical tools used are crucial therefore for obtaining consistent and perhaps reliable results by NGS analysis.

In attempts to simplify the analysis of the enormous amount of NGS data generated, an e-probe based approach was utilized by Stobbe et al. (2014) and Visser et al. (2016). E-probes are pathogen-specific sequences that are rigorously assessed for their specificity and fitness for purpose (Stobbe et al., 2014). When used to screen 18 NGS data sets generated from dsRNA extracted from grapevines, e-probe detection using the program Truffle for data analysis (with e-probes developed for 55 known viruses) was as sensitive as a *de novo* assembly-based NGS data analysis pipeline. In some cases, e-probe detection seemed to be more reliable (Visser et al., 2016).

Cost and complexity are major impediments to the implementation of molecular techniques, but simultaneous detection of various pathogens contributes to cost reduction (Martin et al., 2000; James et al., 2006). The potential to pool samples from different plants, even different plant species, for NGS analysis (Wylie et al., 2012; Villamor et al., 2016; Rott et al., 2017; Verdin et al., 2017) can contribute also to reductions in the time and costs of diagnostics. Also, NGS has the potential to identify novel plant pathogens that may be the causal agents of diseases of unknown etiology that otherwise could not be determined (Kreuze et al., 2009; Barba and Hadidi, 2015; James and Phelan, 2017). Any successful implementation of a new technology for routine use for reliable pest diagnosis requires an understanding of the limitations of the technique. Issues such as the uneven distribution of viruses in plants (Jones et al., 2017) and the fact that RT-PCR was reported to be more reliable than NGS in some cases where viruses or viroids were in low titer (Jo et al., 2018) might indicate the need for caution in implementation and interpretation of NGS results.

## Author Contributions

All authors listed have made a substantial, direct, and intellectual contribution to the work and approved it for publication.

## Conflict of Interest Statement

The authors declare that the research was conducted in the absence of any commercial or financial relationships that could be construed as a potential conflict of interest.
